# Hyperhidrosis and Neurofibromatosis Type 1: A Case Report

**DOI:** 10.7759/cureus.63021

**Published:** 2024-06-24

**Authors:** Rana M Jubran, Samar Sabiq

**Affiliations:** 1 Family Medicine, Ministry of Health, Saudi Arabia, Jeddah, SAU; 2 Consultant Dermatology and Hair Disease, King Abdullah Medical Complex - Jeddah, Jeddah, SAU

**Keywords:** aluminum chloride, case report, saudi arabia, neurofibromatosis type 1, hyperhidrosis

## Abstract

In this report, we present the case of a 20-year-old male with childhood-onset hyperhidrosis affecting his fingers and palm flexor surfaces. Dermatological examination revealed café-au-lait macules, palm and sole involvement, and axillary freckling. A starch-iodine test confirmed localized sweating. Neuroimaging identified neurofibromatosis type 1 (NF1) with subcutaneous nodules and dural ectasia in the thoracic spine. The patient was diagnosed with hyperhidrosis and NF1 based on diagnostic criteria, and he responded well to 20% aluminum chloride for treatment of hyperhidrosis. This case represents a unique occurrence of hyperhidrosis with NF1 in Saudi Arabia. Comprehensive evaluation, including systemic assessment, radiology, and starch-iodine testing, aids in diagnosis and understanding of the underlying mechanisms of this disorder, which remains unexplained.

## Introduction

Hyperhidrosis, a condition affecting the sweat glands, leads to excessive sweat production and exceeds the body’s thermoregulation needs [1]. This condition’s symptom, excessive sweating, significantly impacts patients’ quality of life, disrupts their social and professional engagement, and causes emotional distress [2,3]. Hyperhidrosis can be categorized into primary and secondary types based on its underlying cause [4], affecting 1-3% of the population [5,6]. This study presents the case of a 20-year-old male diagnosed with hyperhidrosis and neurofibromatosis type 1 (NF1) and explores relevant research. In the Kingdom of Saudi Arabia, the link between hyperhidrosis and NF1 remains unidentified.

## Case presentation

A 20-year-old male, previously without any medical history, was presented to our clinic in July 2020 with a long-standing issue of hyperhidrosis affecting his fingers and the flexor surfaces of both arms (palms) since childhood. The hyperhidrosis manifested as excessive sweating without apparent triggers, consistently impacting the same areas with no involvement of other skin sites. These episodes were not accompanied by symptoms like headaches, flushing, hypotension, excessive lacrimation, or salivation. It significantly disrupted the patient’s daily and social life, leading to sleep disturbances. The patient had no history of gastrointestinal or cardiovascular diseases, ear, nose, or throat abnormalities, preexisting parotid gland disorders, or trauma. Additionally, he was not taking any medications, and there was a family history of a similar complaint (his brother).

**Figure 1:**
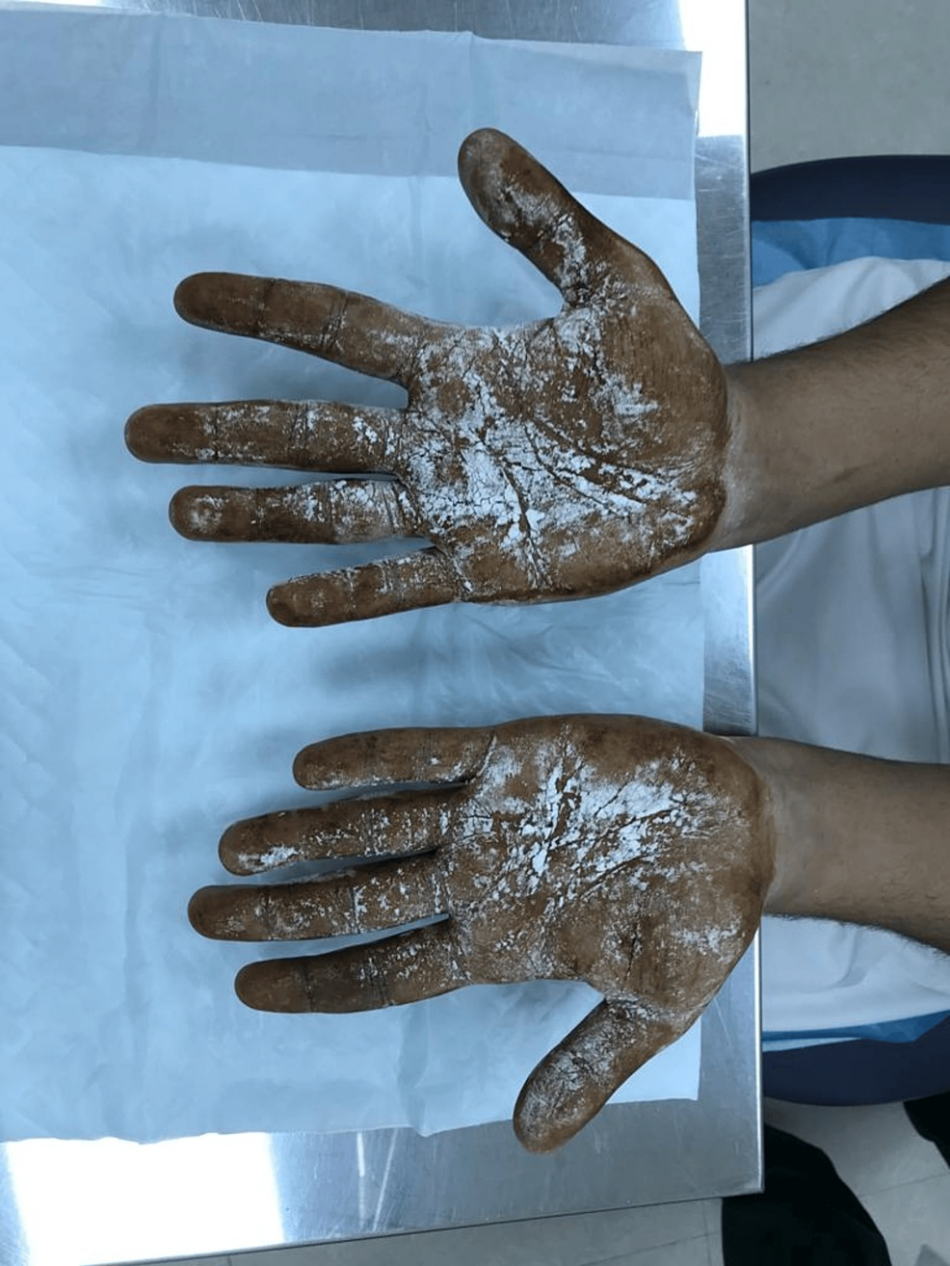
Multiple dark blue areas over the fingers and palms indicate a positive iodine starch test.

Upon dermatological examination, multiple café-au-lait macules and patches measuring 5-15 mm were observed distributed across the body, including the palms and soles. Axillary freckling was also noted during the physical examination. The patient presented with subcutaneous skin-colored nodules on the face and upper limbs, with no hypopigmented lesions or plexiform neurofibromas detected. Furthermore, a starch-iodine test revealed profuse sweating in the well-defined areas of the fingers and the lateral surfaces of both palms shown in Figure 1.

**Figure 2:**
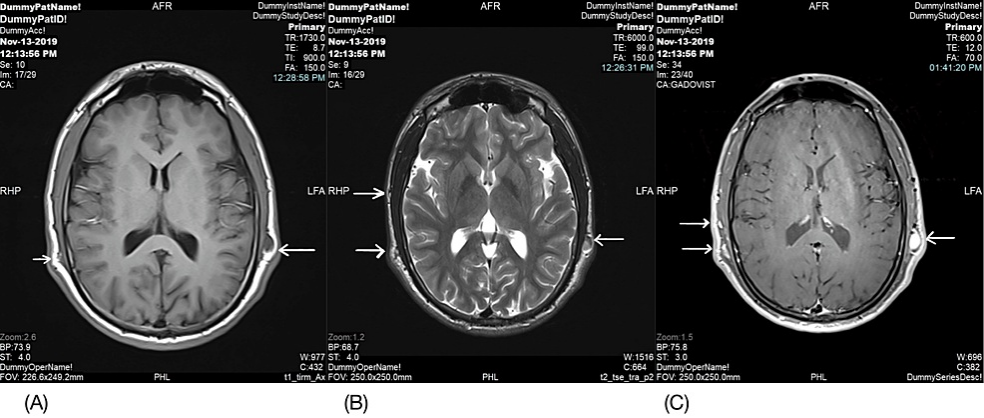
Magnetic resonance imaging of the brain unveiled multiple subcutaneous neurofibromas (arrows)

**Figure 3:**
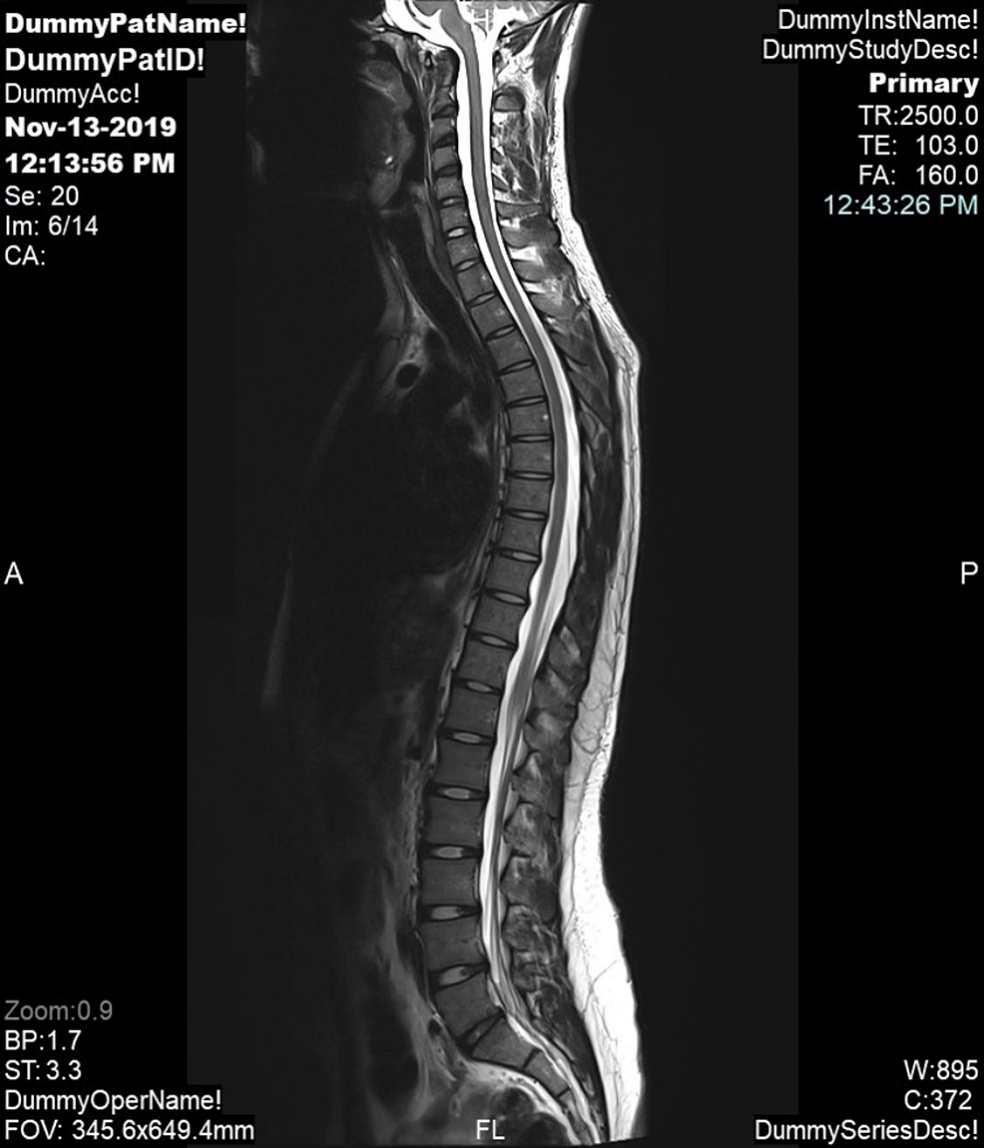
Magnetic resonance imaging of the thoracic spine with dural ectasia

The patient exhibited a normal gait with no facial deformities. Neurological and ophthalmological examinations yielded unremarkable results. Complete blood count, kidney function, and liver function test results all fell within normal ranges. Chest radiography and contrast-enhanced pan-computed tomography did not reveal any abnormalities. However, magnetic resonance imaging of the brain and spine unveiled multiple subcutaneous soft tissue-enhancing nodules shown in Figure 2, which were indicative of neurofibroma and dural ectasia in the thoracic spine shown in Figure 3. Based on the diagnostic criteria for NF and the presence of both cutaneous and neurological features, the patient received a diagnosis of hyperhidrosis and NF1. Notably, our patient responded remarkably well to treatment with 20% aluminum chloride for hyperhidrosis.

We emphasized the importance of maintaining follow-up care to detect any potential new lesions suggestive of NF1 or hyperhidrosis progression. We also scheduled follow-up appointments with the ophthalmology and neurology departments. Unfortunately, the patient did not attend the scheduled follow-up appointments.

## Discussion

Excessive sweating that goes beyond what is biologically necessary is referred to as “hyperhidrosis”. The patient’s excessive sweating in his palms and fingers aligns with primary focal hyperhidrosis, a condition characterized by abnormal sweating not caused by an underlying medical condition or external triggers [7,8].

Based on the distribution of perspiration, the cause of hyperhidrosis may be systemic or localized and classified as primary or secondary [8]. Primary hyperhidrosis often affects the palms and feet and the absence of other symptoms like headaches or flushing further supports a primary diagnosis. Moreover, many cases of hyperhidrosis without a clear related etiology have been documented in the literature and labeled idiopathic [9-12].

A comprehensive evaluation is crucial for patients with hyperhidrosis to rule out systemic conditions that could mimic hyperhidrosis. This evaluation typically involves a detailed medical history, physical examination, and relevant blood tests, such as a complete blood count (CBC), thyroid function test, metabolic panel, and chest X-ray.

Topical aluminum chloride 20% is a well-established first-line treatment for primary focal hyperhidrosis, as used in this case. However, for patients with an inadequate response, alternative options like oxybutynin, iontophoresis, and botulinum toxin injections can be considered [13,14]. Botulinum toxin injections are effective in many cases, with a reported duration of action lasting 6 to 24 months. In cases of secondary hyperhidrosis, treating the underlying medical condition is essential alongside any existing therapy.

Treatment with 20% aluminum chloride resulted in significant improvement in the patient’s hyperhidrosis. We emphasized the importance of maintaining follow-up care to detect any potential new lesions suggestive of NF1 or hyperhidrosis progression.

An autosomal dominant mutation in the NF1 gene, which is found on chromosome 17, results in the condition known as NF1. The diagnosis of NF1 relies on established clinical criteria defined by the National Institutes of Health (NIH). These criteria include having a first-degree relative with NF1, along with cutaneous, ophthalmic, neurologic, and osseous manifestations. Importantly, genetic testing is not always necessary for diagnosis if a patient meets the established NIH clinical criteria. Currently, there is no cure for NF1. Management focuses on regular monitoring by a multidisciplinary team of specialists. such as a dermatologist, neurologist, ophthalmologist, and pediatrician, depending on the patient’s specific needs and medical intervention is only necessary when complications arise. However, regular monitoring allows for early detection and management of potential issues.

A literature review revealed only one documented case report of NF1 associated with hyperhidrosis, and it involved unilateral localized sweating [15]. We were unable to identify a basic brain disorder that could account for the sweating dysregulation [16]. In addition, long-term follow-up for this patient is essential. Monitoring for potential new lesions associated with either NF1 or hyperhidrosis, as advised, will be crucial for optimal management. Additionally, further research investigating the potential association between NF1 and hyperhidrosis would be valuable in expanding our understanding of these conditions.

## Conclusions

We conclude that a thorough examination including a complete history, systemic checkups, radiographic evaluations, and starch-iodine tests is necessary and beneficial in determining the diagnosis and other underlying conditions. Since the etiology of this disorder and the association between hyperhidrosis and NF1 have not yet been explained, presenting the evidence to determine the pathogenesis of the disease will become possible.
